# Pharmacy responses during the COVID-19 pandemic: a questionnaire survey

**DOI:** 10.1186/s12889-022-12589-z

**Published:** 2022-01-25

**Authors:** Shinichi Watanabe, Yu Inami, Hiroshi Kimura, Takaaki Yano, Masafumi Ono, Ryosuke Akizuki, Yukihiro Nawata, Tomomi Tanaka, Kiyoshi Furukawa, Mamoru Tanaka

**Affiliations:** 1 Department of Clinical Pharmacy, College of Pharmaceutical Sciences, Matsuyama University, 4-2 Bunkyo-cho, Matsuyama, Ehime 790-8578 Japan; 2 Ehime Pharmaceutical Association, 7-6-9 Sanban-cho, Matsuyama, Ehime 790-0003 Japan; 3 Department of Pharmacy, Ehime Prefectural Imabari Hospital, 4-5-5 Ishii-cho, Imabari, Ehime 794-0006 Japan; 4 Division of Pharmacy, Ehime University Hospital, 454 Shitsukawa, Toon, 791-0295 Japan; 5 Division of Pharmacy, Ehime Rosai Hospital, 13-27 Minami-komatsubara-cho, Niihama, 792-8550 Japan; 6 Department of Pharmacy, Sumitomo Besshi Hospital, 3-1 Oji-cho, Niihama, 792-8543 Japan; 7 Matsuyama Pharmaceutical Association, 7-6-9 Sanban-cho, Matsuyama, Ehime 790-0003 Japan

**Keywords:** Coronavirus, COVID-19, Disinfection, Ethanol, Infection control, Personal protective equipment, Pharmacy, Pharmaceutical preparations, Physical distancing, Surveys and questionnaires

## Abstract

**Background:**

Coronavirus disease 2019 (COVID-19) has heavily affected the economy, industries, and medicine. Local governments and medical institutions have struggled to respond. The purpose of this questionnaire survey was to evaluate strategies for pharmacy services, availability of ethanol for disinfection, and measures adopted for in-house infection control aiming to enhance future infection control efforts.

**Methods:**

Since pharmacies have been also affected by the COVID-19 pandemic, we surveyed COVID-19 measures taken at 174 pharmacies in Ehime prefecture, Japan.

**Results:**

The survey showed that pharmacies made changes to facilities and equipment, such as installing partitions at dispensing counters, procuring personal protective equipment for employees, and using ethanol for disinfection, even when these items were in short supply. Pharmacies also adopted new strategies, such as holding meetings with suppliers and internal staff via online platforms. Many pharmacies also undertook COVID-19 preventive measures, such as preparing documentation of infection control measures and disinfectants. Moreover, they held lectures and workshops on disinfection and infection control measures.

**Conclusions:**

From public health perspectives, pharmacies should adopt measures to prevent infections spread and, if necessary, utilise online tools and other new strategies to achieve this goal. It is also essential to educate the public about infection control, stockpile supplies, and work with hospitals to prevent COVID-19 spreads.

## Background

Coronavirus disease 2019 (COVID-19) was first confirmed in November 2019 in Wuhan, China, before spreading rapidly worldwide. As of February 2021, the number of infected people had reached over 100 million, with over two million confirmed deaths due to COVID-19. Reports showed that wearing face masks and practicing social distancing were effective means of preventing COVID-19 spreads [1, 2]. In addition, many people have been taking conscious steps to avoid infection.

Medical institutions in Japan have had to deal with COVID-19 in various ways, such as accepting patients with COVID-19 and adopting measures to prevent in-hospital infection. Some medical institutions had to cut back on regular healthcare services. There were significant shortages of ethanol for disinfection and personal protective equipment, such as gloves, gowns, and face masks. Additionally, many medical institutions had difficulty procuring supplies for a brief period. Pharmacies also come into direct contact with patients during medication counselling and dispensing. Therefore, pharmacy staff managed to take extreme care to prevent infection. Although rarely, pharmacists may be directly involved in treatment of patients with COVID-19. Additionally, pharmacists are called upon to provide a variety of other services, such as logistic support for physicians and nurses and procurement and supply of disinfectants. Pharmacists have to be conscious of working to improve public health and control infection among their staff. Pharmacies are visited by patients, pharmaceutical distributors, medical device companies, and other parties. Currently, pharmacies sometimes limit contact with these external parties due to COVID-19. However, there is insufficient information on the approach adopted by individual pharmacies regarding restrictions on meetings and infection control measures, as many pharmacies are unsure of which strategies to adopt. Herein, we aimed to assess the current circumstances and concerns faced by pharmacies in relation to COVID-19. We conducted a questionnaire survey to evaluate strategies for pharmacy services, availability of ethanol for disinfection, and measures adopted for in-house infection control with the aim of enhancing future infection control efforts.

## Methods

**Fig. 1:**
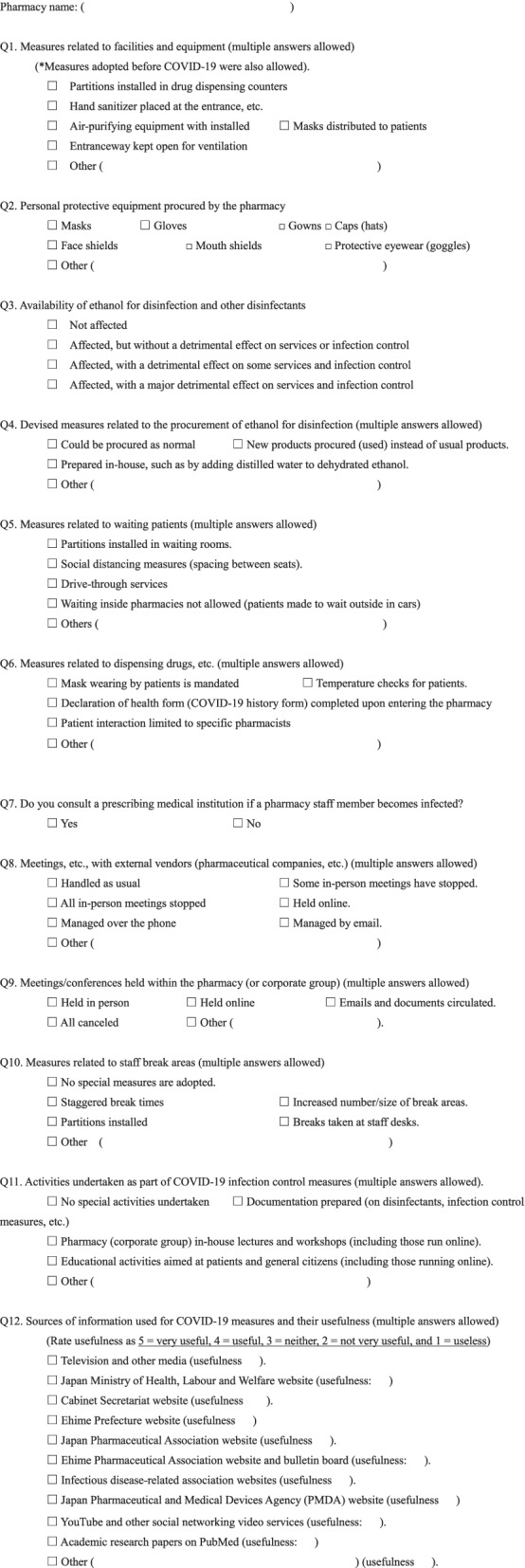
Survey questionnaire. COVID-19, coronavirus 2019

Between 15 September and 10 October 2020, a questionnaire survey on COVID-19-related practices, availability of ethanol for disinfection, and other items was conducted among all dispensing pharmacies in Ehime prefecture, Japan. The questionnaire items are shown in Fig. 1. Instructions were placed on the Ehime Pharmaceutical Association website, and members were notified via e-mails and bulletin board. The website can be accessed by all pharmacies in Ehime Prefecture and information might be exchanged. Questionnaire responses were collected online using Microsoft Forms. The collected data was exported to Microsoft Excel file for calculation and analysis. This analysis was descriptive. Although this research involved human participants, it did not require informed consent. It fell under the category of research that did not use samples or personal information obtained from human participants. Although obtaining informed consent was not necessarily required, information on the content of the research was disclosed and the research participants were given the opportunity to refuse. The respondents did not receive any incentives.

The study was conducted in accordance with the guidelines for human studies adopted by the Ethics Committee of Matsuyama University (Ehime, Japan). This Ethics Committee of Matsuyama University approved the study protocol. The study was also conducted in accordance with the ethical standards laid down in the Declaration of Helsinki, and Ethical Guidelines for Medical and Health Research Involving Human Subjects established by the Ministry of Education, Culture, Sports, Science and Technology and the Ministry of Health, Labor and Welfare of Japan.

## Results

### Measures related to equipment and facilities

**Fig. 2:**
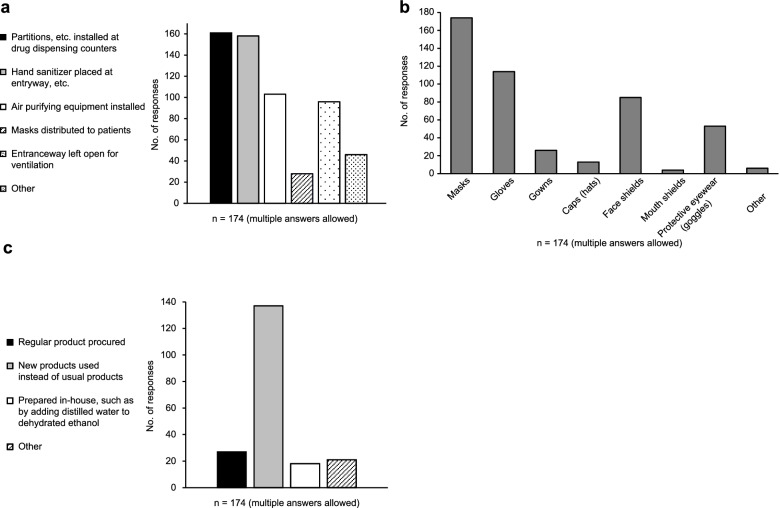
**a**. Measures related to facilities and equipment. **b**. Personal protective equipment procured by pharmacy. **c**. Measures resulting in the procurement of ethanol for disinfection

Out of the 574 pharmacies that are members of the Ehime Pharmaceutical Association, we received valid responses from 174 pharmacies (30.3%). The measures adopted by pharmacies related to equipment and facilities are shown in Fig. 2. Most of these pharmacies introduced new equipment such as safety partitions and plastic curtains at the counters. Other measures adopted by pharmacies included installing air circulators and non-contact thermometers for patients (Fig. 2a).

Personal protective equipment most commonly used by pharmacies included face masks in all pharmacies, gloves, and face shields. Some pharmacies also provided employees with goggles and gowns (Fig. 2b).

Availability of disinfectants, such as ethanol is shown in Table 1.

**Table 1:** Availability of ethanol for disinfection and other disinfectants

	No. of responses (%)
Not affected	11 (6.3)
Affected, but without detrimental effects on services or infection control	142 (81.6)
Affected, with detrimental effects on some services and infection control	19 (10.9)
Affected, with major detrimental effects on services and infection control	2 (1.1)

*n* = 174

Regarding changes in procurement of ethanol for disinfection, many pharmacies used a new product instead of the conventional one, and some pharmacies prepared disinfecting ethanol in-house by adding distilled water to anhydrous ethanol. Other changes included obtaining supplies from the Ehime Pharmaceutical Association and procuring them from different sources, such as online retailers (Fig. 2c).

### Measures related to waiting patients and dispensing drugs

**Fig. 3:**
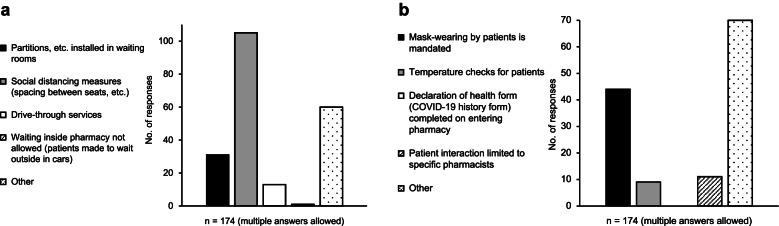
**a**. Measures related to waiting patients. **b**. Measures related to dispensing medication and others

Regarding measures related to waiting patients, socially distanced seating was used in 105 pharmacies. Other measures related to waiting patients included the introduction of cashless payment, removal of books and toys from waiting rooms, hourly disinfecting seating and touched surfaces, and restricting the number of people in pharmacy to a minimum, based on safe floor space (Fig. 3a).

Regarding measures related to dispensing drugs, wearing face masks by patients was required by 44 pharmacies. Other measures related to dispensing drugs included delivering drugs to patients with and without fever separately, using software to provide guidance on drug administration online, and pharmacists providing guidance on drug administration at patient’s car (Fig. 3b).

### Measures adopted for non-patient-facing activities

Regarding measures when a pharmacy staff member became infected, consultation with a prescribing physician in a medical institution was undertaken by 26 pharmacies. The outcomes of this consultation included preventing the infected staff member from attending work for 14 days, suspending high-risk individuals from attending work, seeking staffing support from other pharmacies, closing pharmacy for a limited period, and suspending attendance at work for two weeks if family members would have had returned from pandemic affected regions (Table 2).

**Table 2:** Consultation with a prescribing physician in a medical institution if a pharmacy staff member becomes infected

	No. of responses (%)
Consultation with a prescribing physician in a medical institution	26 (14.9)
No Consultation	148 (85.1)

*n* = 174

**Fig. 4:**
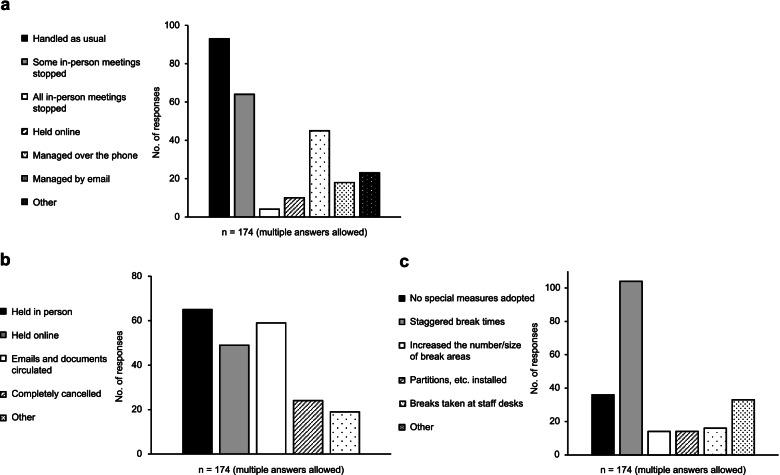
**a**. Meetings and others with external vendors. **b**. Meetings/conferences held within pharmacy (or corporate group). **c**. Measures related to staff break areas

Regarding meetings with external suppliers, these were handled as usual in about half of the pharmacies. Other approaches to meetings with external suppliers included reducing the number of visits and holding meetings across partitions (Fig. 4a).

Regarding in-house meetings/conferences, holding meetings/conferences in person was the most common approach adopted by 65 pharmacies. Other approaches regarding in-house meetings/conferences included reducing their frequency, holding them in larger meeting rooms while practicing social distancing (Fig. 4b).

Regarding changes related to staff break areas, break periods were staggered by about 60% of pharmacies. Other changes related to staff break areas included prohibiting conversation, eating parallel to each other at a fixed distance, disinfecting desks with alcohol after eating, reducing the number of seats, wearing masks when not eating, and installing ventilation fans (Fig. 4c).

**Fig. 5:**
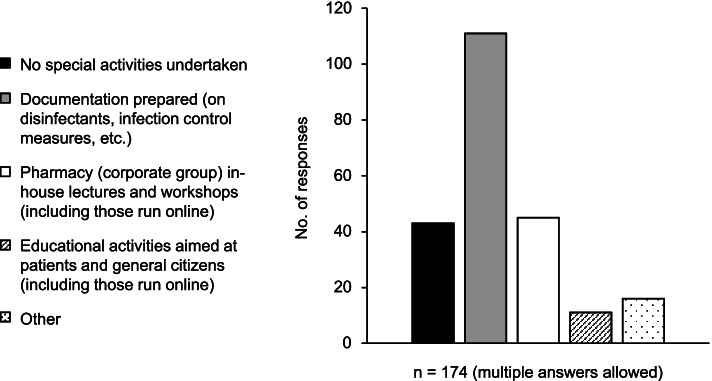
Activities undertaken as part of COVID-19 infection control measures. COVID-19, coronavirus 2019

Regarding activities undertaken as parts of COVID-19 preventive measures, documentation for disinfectants used and infection control measures was prepared by about 60% of pharmacies. Other activities undertaken included establishing in-house infection control teams and providing education on handwashing and wearing face masks in elementary schools (Fig. 5).

### Sources of information used for COVID-19 measures and their usefulness

The most common source of information of COVID-19 measures was the Japan Ministry of Health, Labour, and Welfare website with 140 responses, followed by the Ehime prefecture website with 120 responses, and the Ehime Pharmaceutical Association website with 116 responses. Information sources rated as highly useful included academic literature on PubMed, with 5.00 points, the Ehime Pharmaceutical Association website with 4.26 points, the websites of infectious disease-related associations with 4.24 points, and the Ehime prefecture website with 4.15 points (Table 3).

**Table 3:** Sources of information used for COVID-19 measures and their usefulness

Source of information	No. of responses	Usefulness
Television and other media	116	3.59
Japan Ministry of Health, Labour, and Welfare website	140	4.03
Cabinet Secretariat website	25	3.92
Ehime prefecture website	120	4.15
Japan Pharmaceutical Association website	81	4.01
Ehime Pharmaceutical Association website	116	4.26
Infectious disease-related association websites	38	4.24
Japan Pharmaceutical and Medical Devices Agency	19	4.06
YouTube and other social networking video services	21	3.53
Academic research papers on PubMed, etc.	2	5.00
Other	15	4.07

*n* = 174 (multiple answers allowed)

## Discussion

This study revealed that many participating pharmacies had installed safety partitions and plastic curtains at counters, showing that pharmacies underwent facilities modifications. The increased demand has affected the availability of ethanol for disinfection in many pharmacies. Survey responses showed that pharmacies also adopted measures for waiting patients, such as installing safety partitions in waiting rooms and using drive-through services.

Infection control is important during the COVID-19 pandemic, and pharmacies are also called upon to adopt measures against COVID-19 spreads. According to previous reports, although wearing appropriate personal protective equipment was effective in infection control, pharmacists working at pharmacies would have come into direct contact with patients [3, 4]. Using ethanol for disinfection is effective in preventing transmission of COVID-19 [5, 6], and its demand has grown rapidly during the pandemic. Many pharmacies in this study described using new products instead of their usual products and preparing ethanol in-house for disinfection, such as by adding distilled water to anhydrous ethanol. The World Health Organisation guidelines specify that the concentration of ethanol used for disinfection is 60–80 v/v% [7]. Hence, ensuring an appropriate concentration is important when preparing ethanol in-house for disinfection. This suggests that a flexible approach to obtaining ethanol for disinfection is also important when supplies are limited. It has been reported that ethanol was in short supply not only in Japan, but also in other countries. Moreover, it would be possible to reduce consumption of ethanol disinfectant by effectively washing hands with running water and soap according to the situation [8].

The detrimental effect of COVID-19 on medical institution finances is another concern due to the reduced number of patients and investment in facilities [9]. In addition, shortages of essential facilities and equipment, and loss of personnel must be avoided to ensure the quality of treatment.

Many pharmacies placed some limits on meetings with external suppliers, such as stopping all in-person meetings. Pharmacies also used various methods to obtain information while avoiding in-person meetings, such as online meetings or meetings by phone or e-mail. Online tools are useful for holding meetings and acquiring medical knowledge [10, 11]. Additionally, proactive use of internet tools for interviews and meetings seems to be beneficial. Places of food consumption are prone to airborne droplet dispersal and likely to increase risks of infection. Hence, pharmacies should ideally ensure proper socially distanced seating and avoid unnecessary conversations in these areas [12, 13]. Regarding staff break areas, the survey revealed that many pharmacies introduced staggered staff breaks and increased the size/number of break areas. Some pharmacies undertook educational activities involving patients and general citizens. Since it is epidemiologically beneficial for public health and local residents and general citizens to have accurate information on infection control measures and infectious diseases, pharmacies should ideally be pursuing such activities on regional basis.

Regarding sources of information on COVID-19 measures, commonly cited sources were television and other media, and the Japan Ministry of Health, Labour, and Welfare, which is a public institution. However, the usefulness of television and other media was particularly low. COVID-19 has spread throughout Japan and has detrimentally affected medical care services at many medical institutions. Daily reports on COVID-19 are published in all forms of media, and the internet is awash with vast amounts of information. Media-induced panic about availability of supplies also affected the supply of ethanol and face masks. Many pharmacies accessed the Ehime prefecture website and the Ehime Pharmaceutical Association website and rated these sources as very useful. National information was required. However, local information tended to be more useful. The survey revealed the usefulness of providing local information.

As a limitation, we did not pilot test the questionnaire or test its validity and reliability. The infection situation in Japan was not large, with about 400,000 people infected as of February 2021, or about 0.3% of the population. However, it was true that supplies for infection control were insufficient. It is instructive to note that several pharmacies responded by making ethanol disinfectant, procuring equipment, and using drive-through and online tools. Since flexible responses to epidemic situations are required, further surveys in other regions are considered necessary in the future.

## Conclusions

To maintain medical services and ensure strict infection control under these conditions, pharmacists must maintain an effective supply of disinfectants and provide accurate information on infection control. Although this survey collected information on measures and strategies adopted by pharmacies, more proactive information gathering and sharing is needed to further enhance infection control and maintain healthcare services at individual pharmacies.

## Data Availability

The datasets used and analysed during the current study are available from the corresponding author on reasonable request.

## Abbreviation

COVID-19: Coronavirus disease 2019
